# Interpretable artificial intelligence based on immunoregulation-related genes predicts prognosis and immunotherapy response in lung adenocarcinoma

**DOI:** 10.3389/fbinf.2025.1613761

**Published:** 2025-09-19

**Authors:** Minghao Wang, Yu Wang, Yitong Li, Chengyi Zhang, Canjun Li, Nan Bi

**Affiliations:** 1 Department of Radiotherapy, The First Hospital of China Medical University, Shenyang, China; 2 Department of Radiation Oncology, National Cancer Center/National Clinical Research Center for Cancer/Cancer Hospital, Chinese Academy of Medical Sciences and Peking Union Medical College, Beijing, China; 3 State Key Laboratory of Molecular Oncology, National Cancer Center/National Clinical Research Center for Cancer/Cancer Hospital, Chinese Academy of Medical Sciences and Peking Union Medical College, Beijing, China

**Keywords:** lung adenocarcinoma, immunotherapy efficacy, risk model, single-cell analysis, machine learning, prognosis

## Abstract

**Introduction:**

Lung adenocarcinoma (LUAD) is the most common subtype of non-small cell lung cancer, and its benefit from immune checkpoint inhibitors (ICIs) is controversial, especially for patients without driver gene mutations. The potential of immunoregulation-related genes (IRGs) in predicting the prognosis of LUAD and the efficacy of immunotherapy becomes emerging. There is an urgent need to establish a reliable IRGs-based predictive model of ICI response.

**Methods:**

Extract and merge LUAD RNA sequencing data and clinical data from GEO database. The differences in genomic and tumor microenvironment (TME) cell infiltration landscape between normal lung tissue and tumor tissue were comprehensively analyzed. Unsupervised consistent cluster analysis based on genes related to immune regulation was performed on the samples. ESTIMATE and TIMER algorithms were used to analyze the infiltration of immune cells in different groups, and TIDE score was used to evaluate the effectiveness of immunotherapy. Then, lasso regression was used to establish a prognostic model based on identified key IRGs. XGBoost machine learning algorithm was further developed, with SHapley Additive exPlanations (SHAP) to interpret the model.

**Results:**

The GEO LUAD cohort was divided into two clusters based on IRG expression, with significantly better survival outcomes and immune cell infiltration in the IRG-high group compared to the IRG-low group. TIDE scores indicated that the group with high IRG pattern showed a better response to ICI treatment. Then, we developed an IRG index (IRGI) model based on identified 2 key IRGs, GREM1 and PLAU, and IRGI effectively divided patients into high-risk and low-risk groups, revealing significant differences in prognosis, mutational profiles, and immune cell infiltration in the TME between two groups. Subsequently, the interpretable XBGoost machine learning model established based on IRGs could further improve the predictive performance (AUC = 0.975), and SHAP analysis demonstrated that GREM1 had the greatest impact on the overall prediction.

**Discussion:**

IRGI can be used as a valuable biomarker to predict LUAD patient prognosis and response to ICIs. IRGs play a crucial role in shaping the diversity and complexity of TME cell infiltration, which may provide valuable guidance for ICI treatment decisions for LUAD patients.

## Highlights

- IRGs correlate with patient prognosis and tumor immune cell infiltration
- High/low IRG patterns show distinct mutation profiles and tumor environment
- IRG index (IRGI) risk score predicts outcomes and immunotherapy response
- Interpretable machine learning model on IRGs improves predictive power

## 1 Background

Lung adenocarcinoma (LUAD) is the most common subtype of non-small cell lung cancer (NSCLC), accounting for a significant proportion of cancer-related deaths worldwide (Siegel et al., 2023; Zeng H. et al., 2024). Despite advancements in early detection and targeted treatment, the prognosis for patients with LUAD remains poor, particularly in the advanced stages. Among LUAD patients, those without driver gene mutations represent a particularly challenging patient subgroup, as they lack targeted therapeutic options and may not respond well to chemotherapy (Kim et al., 2019). The advent of immune checkpoint inhibitors (ICIs) has revolutionized the treatment landscape for LUAD, demonstrating significant clinical benefits in some patients (Wang et al., 2023). However, patient responses to ICIs are highly heterogeneous, with only a subset of patients achieving durable responses, which highlights the urgent need for predictive biomarkers that can effectively differentiate between patients most likely to benefit from ICI and those may require alternative treatment strategies.

Recent research has demonstrated that immunoregulation-related genes (IRGs) played a pivotal role in the complex interplay between the tumor and the immune system (Chen Y. et al., 2021). They modulate tumor microenvironment (TME) and influence the balance between immune activation and suppression, and their expression levels can serve as early predictors of prognosis and response to immunotherapy (Nahar et al., 2018; Usó et al., 2017; Wang et al., 2024). In the context of LUAD, particularly in patients without driver gene mutations, IRGs could hold the key to unlocking the potential of immunotherapy and guiding more personalized treatment approaches.

The GTPase of immunity-associated protein (GIMAP) family genes, for instance, have been implicated in the tumorigenesis of LUAD and associated with immune cell infiltration and immune checkpoint molecules (Zhang et al., 2024; Moreira et al., 2023). Their expression levels in tumor tissue were significantly correlated with overall survival, suggesting their potential as prognostic biomarkers and predictors of immunotherapy response (Qin et al., 2022; Huang et al., 2016). Moreover, the development of IRG prognostic index has revealed the ability to predict patient prognosis and response to immunotherapy, reflecting the complex interactions within the TME and the diverse immunological characteristics of LUAD (Liu et al., 2023; He et al., 2023). In this study, we aim to provide an overview of the current understanding of IRGs for LUAD patients, focusing on their role in early prediction of patient prognosis and response to immunotherapy. We further discuss the biological mechanisms by which these genes influence tumor immunity, and the potential for integrating IRG-related biomarkers into the clinical practice to facilitate personalized therapeutic decision-making.

## 2 Methods

The messenger RNA (mRNA) data and corresponding clinical parameters of a cohort comprising 226 normal samples and 642 tumor samples from LUAD patients were extracted from the Gene Expression Omnibus (GEO) database. Among these, samples with missing survival information or an overall survival (OS) of less than 1 month were excluded. Immune-related genes were identified using Weighted Gene Co-expression Network Analysis (WGCNA), Cytoscape and other methods, followed by unsupervised consensus clustering based on IRGs. The ESTIMATE and TIMER algorithms were employed to analyze immune cell infiltration across different groups, and Tumor Immune Dysfunction and Exclusion (TIDE) scores were deployed to predict the efficacy of immunotherapy (Chi et al., 2020; Zeng D. et al., 2024). Subsequently, a prognostic model of genes associated with patient outcomes was constructed using a least absolute shrinkage and selection operator (LASSO) Cox regression model. This model was independently validated in both the TCGA dataset and immunotherapy datasets, with potential therapeutic drugs identified. Finally, we used the XGBoost machine learning algorithm to evaluate the importance of variables, with SHapley Additive exPlanations (SHAP) employed to interpret the model.

### 2.1 Collection of database data

A systematic search of the GEO database for LUAD gene expression data was conducted. Our analysis included four LUAD expression profile cohorts: GSE10072, GSE32863, GSE40791, and GSE68465 (Landi et al., 2008; Shedden et al., 2008; Selamat et al., 2012; Zhang et al., 2012). We downloaded the matrix files for each GEO cohort for further analysis. All acquired RNA expression profiles were normalized using log2 (TPM +1) transformation. In total, we included 654 LUAD tumor tissue samples and 226 normal samples. To correct for batch effects caused by non-biological technical variation, we used the ComBat algorithm from the svaR package.

### 2.2 Functional analyses

Differentially expressed genes (DEGs) between normal tissues and LUAD samples were identified using empirical Bayes methods in the limma R package (Ritchie et al., 2015). Significant DEGs were identified based on the cutoff of P < 0.05 and a dynamic threshold of |logFC| ≥ 0.828 (calculated as |logFC| ≥ [mean(|logFC|) + 2sd(|logFC|)]) (Rong et al., 2020). Additionally, we performed a protein-protein interaction (PPI) analysis using the STRING database (https://cn.string-db.org) to explore the relationships among the DEGs. The PPI network was constructed with Cytoscape software, and key hub genes were identified by calculating Degree scores using the CytoHubba plugin (Shannon et al., 2003). Gene Ontology and Kyoto Encyclopedia of Genes and Genomes (GO/KEGG) pathway enrichment analyses were deployed to identify the potential functions of DEGs.

### 2.3 Consensus clustering

By utilizing the ConsensusClusterPlus (Wilkerson and Hayes, 2010), an unsupervised consensus clustering analysis was performed to investigate the expression profile data of prognostic genes. The optimal number of clusters was determined based on the cumulative distribution curve, and the process was repeated 1,000 times to ensure the stability of the results. We have uploaded the complete code and parameter settings of ComBat and ConsensusClusterPlus to GitHub. For more details, please visit: https://github.com/mikelu1997/consensus.

### 2.4 Tumor immune microenvironment

Multiple algorithms, including ESTIMATE (Liu et al., 2020) and TIMER (Zhou et al., 2020), were employed to analyze the tumor immune microenvironment. The ESTIMATE algorithm was used to evaluate ESTIMATE scores, immune scores, and stromal scores, while the TIMER algorithms was utilized to assess the abundance of immune cell infiltration across different categories.

### 2.5 Prediction of immunotherapy response

The TIDE score is an online tool (http://tide.dfci.harvard.edu/) designed to evaluate the efficacy of immunotherapy in different risk groups and assess the likelihood of tumor immune evasion (Wang et al., 2022). A higher TIDE score indicates poorer response to ICIs. In this study, we identified LCN2, MUC4, CDH17, COMP, GREM1, COL11A1, MMP9, THBS2, COL1A2, COL3A1, PLAU, CNTF, CXCL13, CXCL9, and CCL19 genes were associated with response to ICIs (Chen D. et al., 2021). We further extracted the expression values of these 15 genes to analyze the expression patterns of immune checkpoint-related genes in different groups.

### 2.6 Construction and validation of IRG-related risk signature

We developed a RiskScore signature to comprehensively evaluate the role of IRGs in patient prognosis and response to immunotherapy. The glmnet R package was used to perform LASSO-Cox regression analysis with 10-fold cross-validation. Ultimately, a linear equation for immunoregulation-related gene index (IRGI) was constructed to predict overall survival (OS) of early-stage LUAD patients: RiskScore = [coef (Siegel et al., 2023) × GeneExp (Siegel et al., 2023)] + [coef (Zeng H. et al., 2024) × GeneExp (Zeng H. et al., 2024)] + …+ [coef(i) × GeneExp(i)] (Zhou et al., 2019). Kaplan-Meier curves were generated using the survival and survminer R packages to conduct prognostic analysis and to evaluate 2-year, 3-year, and 4-year survival rates in the test cohort. To validate the effect of IRGI risk model in predicting patient prognosis and therapeutic response to immunotherapy, external datasets, including GSE72094, the Cho cohort (Cho et al., 2020), and VanAllen cohort (Van Allen et al., 2015), were utilized.

### 2.7 Single-cell RNA sequencing analysis

The GSE229353 (Hui et al., 2023) data set includes single-cell RNA-sequencing (scRNA-seq) data from six LUAD samples treated with immunotherapy. Quality control procedures were applied to filter single cells based on the following criteria: 1) Each gene was expressed in more than 200 genes and in more than 3 cells; 2) The number of genes expressed in a cell ranged from 500 to 50,000; 3) The percentage of mitochondrial genes in a single cell was less than 15% (Yates et al., 2025). After applying these filters, a total of 15,293 cells were retained. The SEURAT R package was used to process the scRNA-seq data. To eliminate batch effects across the six samples, the “FindIntegrationAnchors” and “IntegrateData” functions were applied. Next, the “ScaleData” function was used to scale scRNA-seq data, and principal component analysis (PCA) was performed to reduce dimensionality. Moreover, “FindClusters” and “FindNeighbors” functions were executed with parameters set to dim = 9 and resolution = 0.1 to cluster the single cells into different subgroups. We obtained cell markers for different cell types from CellMarker 2.0 (Hu et al., 2023) and used these markers to annotate the single cells. Finally, single cells were visualized using t-SNE plot generated by the “RunTSNE” function.

### 2.8 Interpretable machine learning

The XGBoost model-related packages were loaded in R, and data was divided into training (70%) and validation (30%) sets using a stratified random sampling method. The XGBoost machine learning model was trained on the training set, and the predictive performance was evaluated using area under the curve (AUC) values. To achieve a deeper understanding of the contribution of each feature in the model prediction, we applied SHAP to interpret the XGBoost model. The shap package was used to calculate the contribution values of each feature for individual samples. SHAP value analysis was conducted to rank the importance of each feature and identify its directional impact within the model. Furthermore, we generated feature importance plots, dependence plots, and force plots to visually represent the SHAP analysis results.

### 2.9 Quantitative reverse transcription PCR (qRT-qPCR)

Total RNA was extracted from normal human lung epithelial cells (BEAS-2B) and A549 lung adenocarcinoma cell lines using Trizol (Thermo Fisher Scientific, Sweden) (all cell lines were maintained according to the supplier’s recommendations). Reverse transcription was performed according to the instructions of the Takara Kit (Takara, Maebashi, Japan). SYBR Green Premix Ex Taq kit (Takara) was used to quantitatively detect PLAU and GRME1 in normal cells and lung adenocarcinoma cells. QRT-PCR was performed using the Roche Applied Science Light Cycler 480 instrument. The cycle threshold (CT) (2–△△CT) method was employed to calculate the data. The normalized expression levels were compared with those of β-actin using the comparative CT method. The primers used in this study are presented in Supplementary Table S3.

### 2.10 Statistical analysis

All data calculations and statistical analyses were performed using R programming (version 4.2.1). The Kaplan-Meier method was applied for survival analysis, and the predictive performance of the risk model was evaluated using time-dependent receiver operating characteristic (ROC) curves via the timeROC package. For comparisons between two groups of continuous variables, independent Student’s t-tests were used to analyze normally distributed variables, while the Mann-Whitney U test was employed for non-normally distributed variables. All two-sided statistical p-values less than 0.05 were considered statistically significant.

## 3 Results

### 3.1 Identification of DEGs and functional pathways regulating tumor immune microenvironment

**FIGURE 1:**
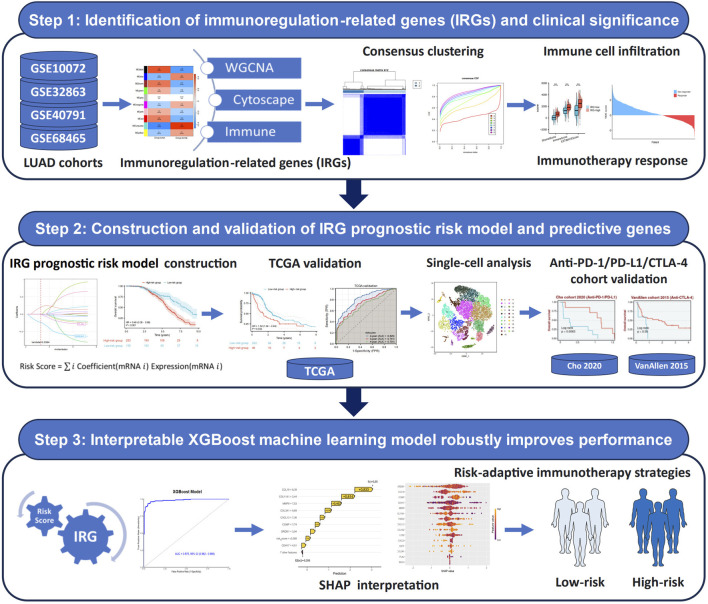
Flow chart of this study.

**FIGURE 2:**
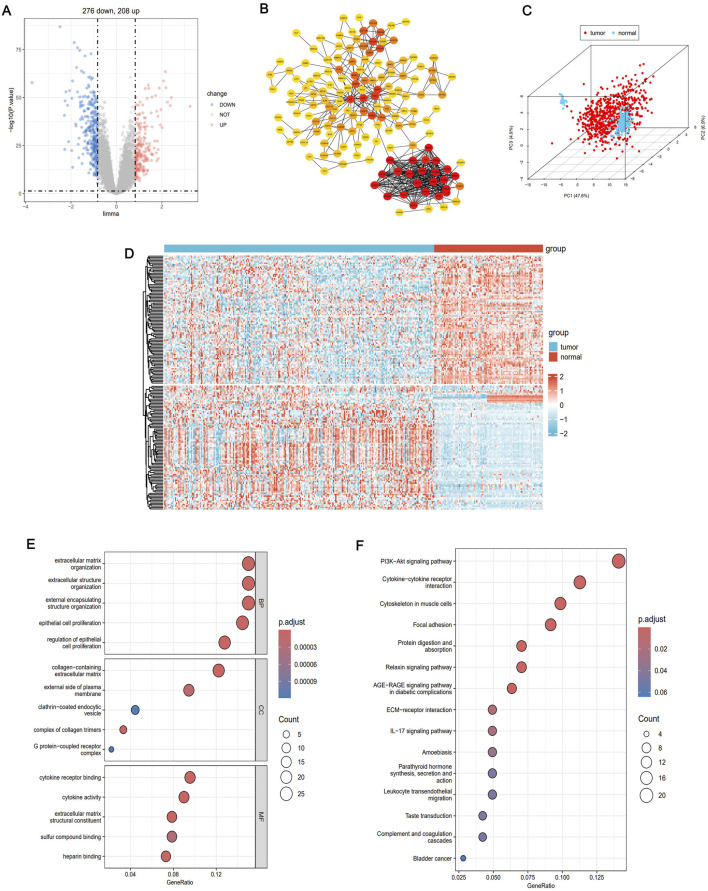
Identifies LUAD genes related to TME regulation. **(A)** Volcano atlas showed differentially expressed genes between normal group and LUAD group (|FC| >0.83, P < 0.05). **(B)** Constructing protein-protein interaction (PPI) network between differentially expressed genes using STRING database. **(C)** Principal component analysis (PcoA) of key genes revealed two completely disjoint populations, indicating that these key genes could well distinguish tumor tissue from normal tissue. **(D)** Heat maps showed the differential expression patterns of key genes in tumor tissue and normal tissue. **(E)** GO enrichment analysis of differentially expressed genes. **(F)** KEGG enrichment analysis of differentially expressed genes.

The flow chart of this research is presented in Figure 1. First, to explore DEGs and their functional roles in regulating TME, we integrated four publicly available datasets in LUAD: GSE10072, GSE32863, GSE40791, and GSE68465. A comprehensive comparison of gene expression between normal and tumor samples determined 484 DEGs, consisting of 276 downregulated and 208 upregulated genes (Figure 2A). To further identify key genes that regulate TME, we constructed a PPI network using the STRING database (network type: physical subnetwork, minimum required interaction score: 0.4) and Cytoscape software (Cytohubba plugin, Degree >15). This analysis identified a core PPI network comprising 178 genes (Figure 2B). These genes were hypothesized to be involved in the regulation of TME and LUAD progression. To assess whether these crucial genes could distinguish between LUAD and normal tissue, we performed PCA to reduce the dimensionality of the gene expression data. The PCA results revealed two completely separate clusters, with normal samples and LUAD tumor samples forming distinct groups, suggesting significant differences in the expression patterns of key genes between the two groups (Figure 2C). A heatmap of these genes further emphasized their differential expression patterns across the samples (Figure 2D). Moreover, we conducted GO and KEGG enrichment analyses on the identified genes to uncover their biological functions. The enriched results revealed that the DEGs were significantly associated with immune response, metabolic processes, cell signaling, and cell proliferation and regulation (Figures 2E,F). These findings suggest that the identified DEGs may play pivotal roles in immune regulation and tumor progression, thereby highlighting their potential as therapeutic targets or biomarkers in LUAD.

### 3.2 WGCNA detection of tumor-related modules and identification of key IRGs

**FIGURE 3:**
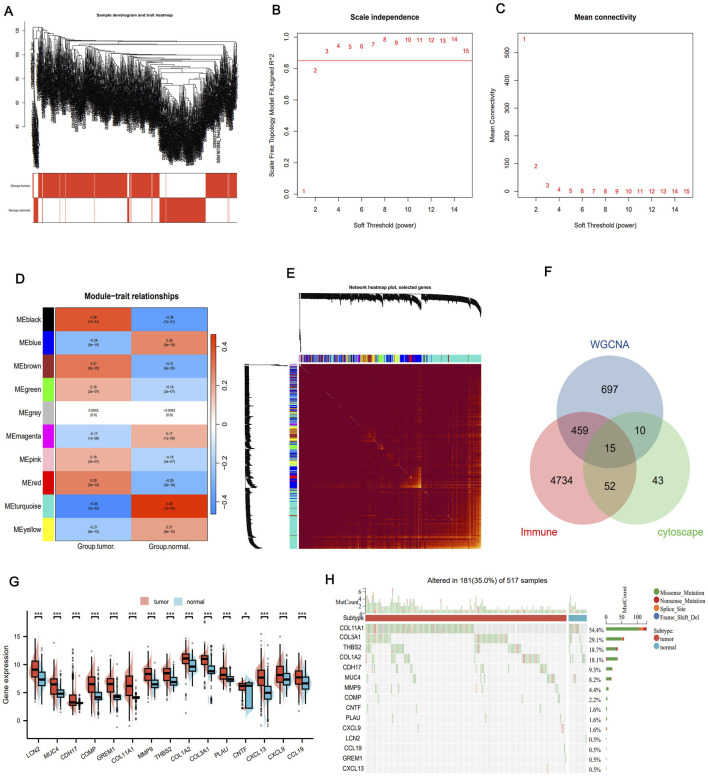
WGANA detection immune-related module. **(A)** Sample clustering trees and trait heat maps based on GEO transcriptome data present tumor-related modules. **(B)** Analysis of the scale-free fit index of the various soft threshold capabilities and the average connectivity of the various soft threshold capabilities. **(C)** Heat maps identify associated feature genomes called meta modules. **(D)** Thermal maps of the LUAD module and clinical features. **(E)** TOM maps of all filtered genes based on their co-expression relationships are visualized by heat maps. **(F)** Venn diagrams showing common genes where key module genes, DEG and immune-related genes intersect. **(G)** Differential expression of IRGs between tumor and normal tissues. **(H)** Mutational landscape of genes associated with immune activation. *P < 0.05, **P < 0.01, ***P < 0.001.

We performed WGCNA to further identify co-expression modules and determine immune-regulatory key genes related to LUAD progression. A co-expression network was constructed based on mRNA expression data, which allowed to uncover tumor-associated modules and genes (Figure 3A). To construct the scale-free network, the optimal soft-thresholding power was determined to be β = 3, as evidenced by the analysis of network topology (Figures 3B,C). The co-expression network revealed a total of 10 distinct modules. Notably, the black module exhibited the strongest correlation with immune clusters (r = 0.38, P = 7e-31, Figure 3D). This module contained 1,181 genes that significantly correlated with tumor biology (Supplementary Table S1). To further visualize the gene relationships within the black module, we generated a Topological Overlap Matrix (TOM) heatmap, which illustrated the degree of association and co-expression between genes (Figure 3E). Next, we intersected the DEGs, WGCNA tumor-related modules, and immune-related genes from the ImmPort database (https://www.immport.org/shared/) (Xin et al., 2021), leading to the identification of 15 candidate IRGs (Figure 3F). These genes were significant upregulation in LUAD tumor samples compared to normal tissues (Figure 3G). Finally, we investigated the mutation landscape of these IRGs. Among the 517 LUAD samples, 35.0% of the patients exhibited mutations in at least one of the key genes. COL11A1 was the most frequently occurred mutation, followed by COL3A1, THBS2,COL1A2, CDH17, MUC4, MMP9, and COMP (Figure 3H). These findings highlight the pivotal role of these IRGs in LUAD, including their prognostic value as immune regulatory factors in cancer biology, and their predictive effect for immunotherapy efficacy.

### 3.3 Consensus clustering analysis of immune-related gene expression

**FIGURE 4:**
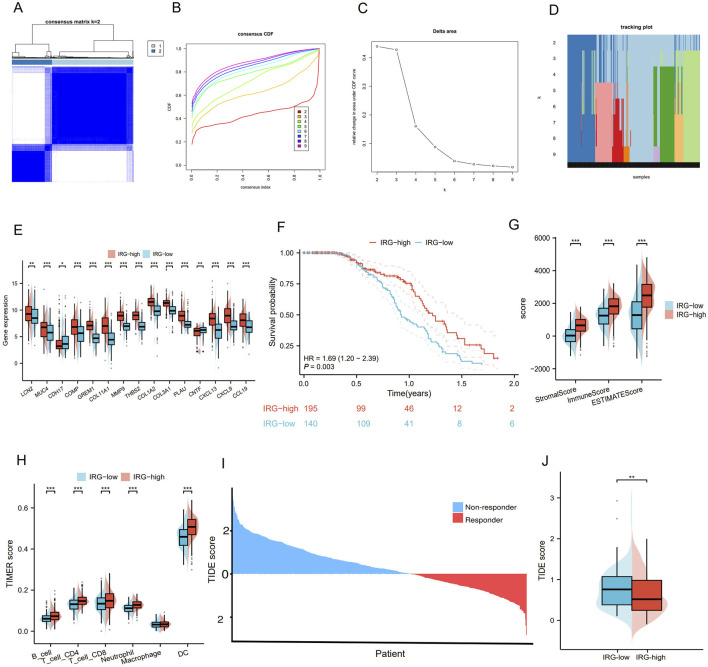
Subtype identification, survival analysis and tumor immune microenvironment analysis. **(A)** Consensus clustering shows that 2 clusters are the most stable. **(B–D)** Consensus clustering model, using the cumulative distribution function (CDF), with k values from 2 to 9. **(E)** Expression of IRG in both modes. **(F)** The difference in prognosis between the two models. **(G)** ESTIMATE score, immune score and matrix score for both models. **(H)** Abundance of immune cell infiltration calculated by TIMER algorithm. **(I)** TIDE analysis bar plot and heatmap. **(J)** TIDE scores for IRG-high and IRG-low groups.

In order to investigate the relationship between immune-related prognostic genes and LUAD subtypes, we performed consensus clustering analysis. Based on the cumulative distribution function (CDF) values, we classified LUAD patients into two distinct clusters (k = 2, Figures 4A–D). Cluster 1 (n = 438) exhibited high expression levels of these immune-related genes and was defined as the IRG-high pattern, while Cluster 2 (n = 215) displayed low expression levels, categorized as the IRG-low pattern (Figure 4E). Notably, survival analysis revealed a significant survival difference between the two patterns. The IRG-high pattern was associated with a more favorable prognosis, while the IRG-low pattern was linked to poorer survival outcomes (Figure 4F). These results suggest that the expression levels of immune-related genes play a crucial role in influencing the prognosis of LUAD patients and could serve as a potential biomarker for patient stratification and therapeutictargeting.

### 3.4 Tumor microenvironment landscape in two molecular patterns

We utilized the ESTIMATE algorithm to compare the immune microenvironment between the two molecular patterns. The results demonstrated that the IRG-high model exhibited higher ESTIMATE, immune, and stromal scores compared to the IRG-low model (Figure 4G). Additionally, according to TIMER scores, the IRG-high pattern was associated with increased abundance of immune cells, including B cells, CD4^+^ T cells, CD8^+^ T cells, neutrophils, and dendritic cells (DC), as shown in Figure 4H. TIDE scores were also calculated to assess the potential clinical efficacy of immune therapy in different risk groups (Fu et al., 2020). TIDE is a computational tool that reflects the potential for tumor immune evasion. Higher TIDE scores are generally associated with poorer efficacy of ICIs. In this study, we revealed that the IRG-low pattern had significantly higher TIDE scores compared to the IRG-high pattern, suggesting that patients in the IRG-high group might benefit more from ICI therapy (Figures 4I,J). These findings underscore the differences in the tumor immune microenvironment between the IRG-low and high groups, as well as their impact in determining the prognosis of LUAD patients and their potential response to immunotherapy. The differential TIDE scores further highlight the potential for personalized treatment strategies based on immune-related molecular patterns.

### 3.5 Construction of immunoregulation related gene index risk model

**FIGURE 5:**
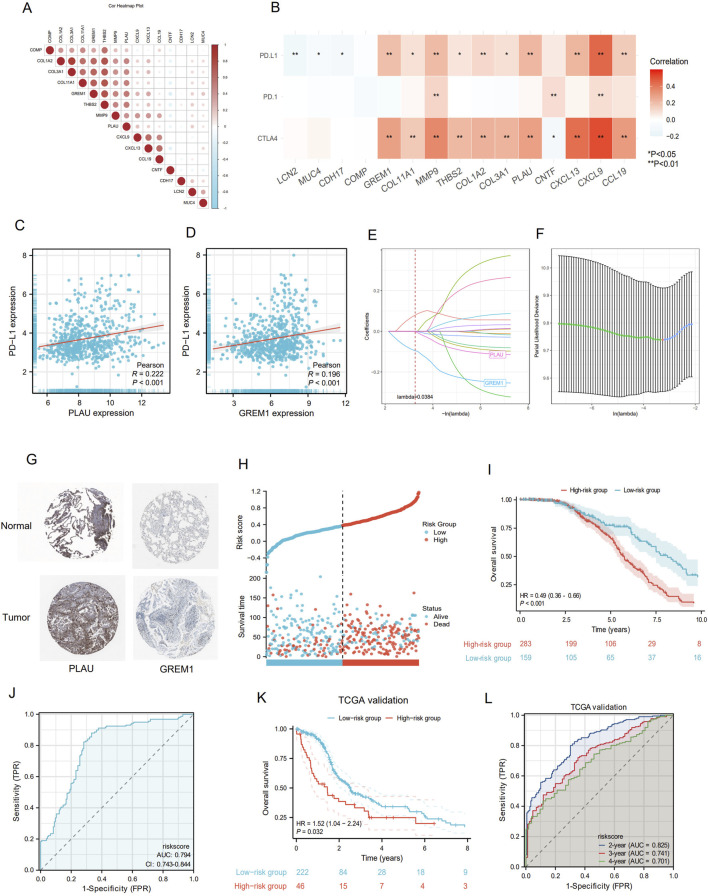
Construction of prognostic signature by IRGI. **(A)** Spearman was used to analyze the relationship among 15 IRGs. Negative correlation is blue and positive correlation is red. **(B)** Correlation of 15 IRGs with immune checkpoint molecules. **(C)** Correlation between PLAU expression and PD-L1 expression. **(D)** The correlation between GREM1 expression and PD-L1 expression. **(E,F)** LASSO-Cox regression analysis based on 15 prognostic genes. **(G)** The immunohistochemical staining results revealed significant differences of key molecules at the protein expression between normal and tumor tissues. **(H)** Proportion of deaths in high and low risk groups as RiskScore values increased. **(I)** Prognostic analysis of two risk groups in the GEO LUAD cohort. **(J)** ROC curve analysis of GEO LUAD cohort risk model over time. **(K)** Prognostic analysis of two risk groups in the TCGA LUAD cohort. **(L)** Time-dependent ROC analysis of the TCGA LUAD cohort.

Using Spearman correlation analysis, we observed that most of the IRGs exhibited positive correlations with each other (Figure 5A). Furthermore, expression of some molecules, such as GREM1, MMP9, PLAU, CXCL13, CXCL9, and CCL19, was positively correlated with immune checkpoint molecules, especially programmed cell death-ligand 1 (PD-L1) and cytotoxic T-lymphocyte associated protein-4 (CTLA-4), with PLAU and GREM1 showing particularly significant correlations (Figures 5B–D). Based on the results from LASSO-Cox regression analysis (Figures 5E,F), we selected PLAU and GREM1 as the optimal indicators for constructing the prediction model. GO/KEGG analysis indicated that both PLAU and GREM1 were enriched in the pathways of immune response regulation, immune cell infiltration, and the remodeling of the extracellular matrix. PLAU was involved in angiogenesis, cell migration and invasion, and GREM1 was associated with the transforming growth factor (TGF)-beta signaling pathway, which was important for tumor progression and immune evasion (Supplementary Figure S1). We also explored single nucleotide variants and copy number variations correlation with the targeted PLAU and GREM1 gene expression, and both high PLAU and GREM1 expression was significantly associated with more TP53, CSMD3, and LRP1B mutations (Supplementary Figure S2). Upon reviewing the Human Protein Atlas (HPA) database, we analyzed immunohistochemistry (IHC) data for these key molecules and qualitatively observed obvious expression differences between normal and LUAD samples (Figure 5G).

Furthermore, we integrated four databases, including hTFtarget, ENCODE, GTRD and ChIP-Atlas, and identified five upstream regulatory transcription factors, namely CTCF, EP300, MAX, RAD21, and KDM4A, which are common to both PLAU and GREM1(Supplementary Figure S3). The risk score was calculated as follows: RiskScore = (−0.095) × (GREM1 expression) + (−0.002) × (PLAU expression). These results were then incorporated into the IRGI risk model. We calculated the IRGI risk score for each patient in the LUAD dataset based on the expression levels and risk coefficients of two key IRGs (PLAU and GREM1; Figure 5H). Low-risk patients had significantly longer OS than high-risk patients (P < 0.001, hazard ratio [HR] = 0.49 [0.36–0.66]; Figure 5I). The ROC curve indicated the accuracy of this model, yielding an AUC value of 0.794 (95% confidence interval [CI]: 0.743–0.844) (Figure 5J). Furthermore, in the TCGA validation cohort, we confirmed OS of low-risk patients was significantly higher than that of high-risk patients (p = 0.032, HR = 1.52 [1.04–2.24]) (Figure 5K). To further evaluate the efficacy of this model, we utilized time-dependent ROC curves, which demonstrated good predictive ability of this IRGI risk model over a 4-year period (Figure 5L). We also performed chemotherapeutic drug predictions for patients with LUAD in the high-risk and low-risk groups to provide optimal treatment regimens. Cisplatin, Cyclophosphamide, Gemcitabine, Paclitaxel, Vinorelbine and Doceaxel were more effective for the high-risk group (Supplementary Figure S4).

### 3.6 Validation of IRGI risk model in immune cohorts from GEO, external databases, and single-cell analysis

**FIGURE 6:**
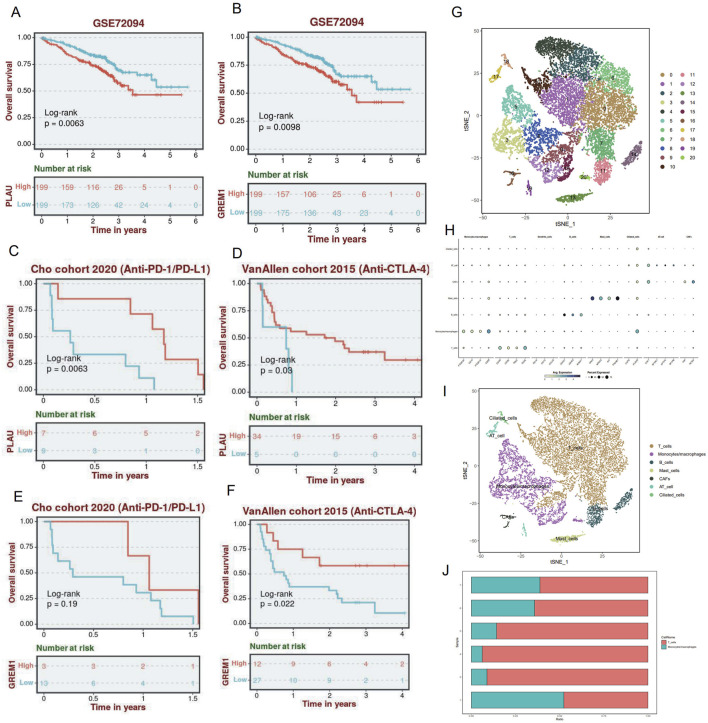
Validation of external database data and Single-Cell analysis of single-cell sequencing data analysis. **(A,B)** Prognostic analysis of PLAU and GREM1 risk groups in the GSE72094 validation cohort. **(C–F)** In Cho and VanAllen cohorts, PLAU and GREM1 both effectively predicted the therapeutic efficacy of ICI. **(G)** t-SNE diagram after cell clustering. **(H)** Expression of marker genes in 7 cell types. **(I)** The distribution of 7 cell types is shown in the t-SNE diagram. **(J)** The ratio of mononuclear/macrophages to T cells (CD4^+^ and CD8^+^ T cells) in the six samples.

To validate the predictive value of the IRGI risk model in ICI therapy, we applied independent GSE and external datasets. In the GSE72094 validation cohort, patients with high expression of PLAU (P = 0.006; Figure 6A) and GREM1 (P < 0.001; Figure 6B) showed significantly worse OS compared to patients with low expression of these genes. However, when evaluating ICI therapy response in the Cho and VanAllen cohorts, high expression of PLAU and GREM1 was significantly associated with better survival outcomes, suggesting that these genes might be predictive of improved efficacy of anti-programmed death 1 (PD-1)/PD-L1 and anti-CTLA-4 therapy (Figures 6C–F). Further validation was carried out using the scRNA-seq data from GSE229353, which included 6 NSCLC patients who underwent neoadjuvant chemotherapy or combination immunotherapy with chemotherapy. A total of 26,930 single cells were initially obtained, with 15,293 cells remaining after stringent quality control procedures for subsequent analysis. The gene expression levels were normalized, and the single cells were clustered into 20 distinct clusters (Figure 6G). Moreover, based on markers from CellMarker2.0, the clusters were categorized into 7 cell types (Figures 6H–J; Supplementary Table S2). The majority of the cells were identified as monocytes/macrophages and T-cells (both CD4^+^ and CD8^+^ T-cells), which are key participants in the immune response. These findings support that ICI therapy leads to increased T-cell infiltration and a better immune response, further corroborating the potential of the IRGI risk model in predicting the efficacy of immunotherapy.

### 3.7 Interpretable XGBoost machine learning model on IRGs with improved predictive performance

**FIGURE 7:**
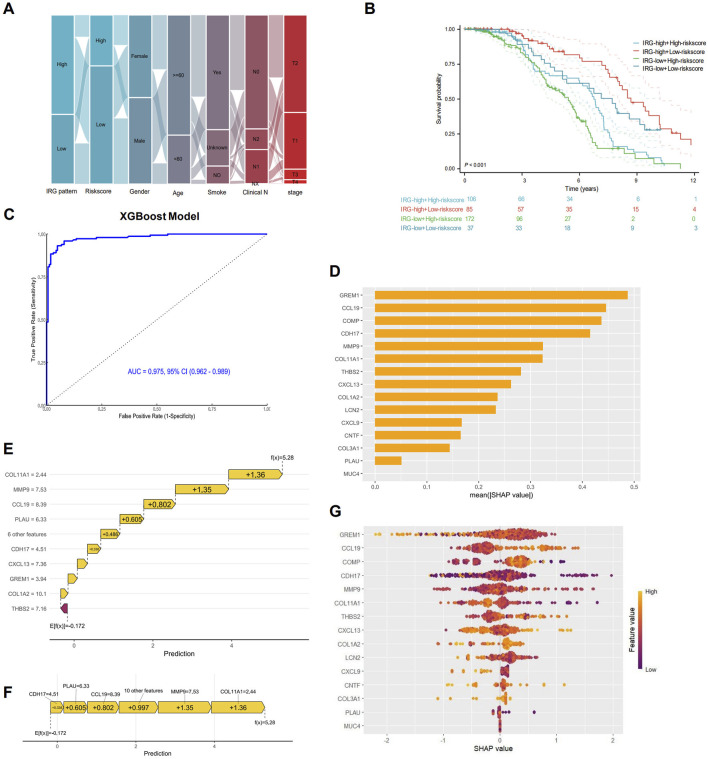
Interpretable XGBoost machine learning model construction. **(A)** Sankey maps based on IRG pattern, risk score, sex, age, smoking, and pathological stage. **(B)** Survival analysis based on IRG signature patterns and risk scores. **(C)** Receiver operating characteristic (ROC) curve for the predictive effect of IRG-based XGBoost machine learning model. **(D)** Bar plot showing the mean SHapley Additive exPlanations (SHAP) values for each IRG in the XGBoost model. IRGs are ranked by their relative importance in the overall model. SHAP waterfall plot **(E)** and force plot **(F)** showing the contribution of each IRG to the overall XGBoost model prediction. **(G)** SHAP beeswarm plot illustrating the impact of each IRG on the overall prediction. IRGs are ranked by their mean SHAP values, with points colored according to feature values (red for high, blue for low).

Sankey diagram showing the distribution of patients across different characteristics, and most of patients with the IRG-high pattern had IRGI low-risk score (Figure 7A). The combined model integrating IRG signature patterns with IRGI risk score also confirmed that patients with IRG-high pattern and IRGI low-risk score exhibited the significantly best survival, while patients with IRG-low pattern and IRGI high-risk score had the worst survival outcomes (P < 0.001; Figure 7B). To further improve the predictive effect, we developed XGBoost machine learning predictive model using pre-identified IRGs, and AUC value of the XGBoost model was strikingly improved to 0.975 (95% CI, 0.962–0.989; Figure 7C). SHAP analysis indicated that GREM1, CCL19, COMP genes ranked in the top three important contributions to the XGBoost model predictions (Figure 7D). The impact of each IRG feature on the prediction is illustrated in SHAP waterfall and force plots (Figures 7E,F). With the basal prediction of −0.172 in the XGBoost model, COL11A1, MMP9, CCL19, and PLAU increased the overall prediction by +1.36, +1.35, +0.802, and +0.605, respectively (Figures 7E,F). SHAP importance scatter plot demonstrated the SHAP values and feature values for a specific IRG (Figure 7G). Each dot represents a patient, and the color indicates the feature value. GREM1 had the highest median SHAP values, indicating the greatest impact on the overall prediction.

### 3.8 The mRNA expression levels of prognosis-related genes

**FIGURE 8:**
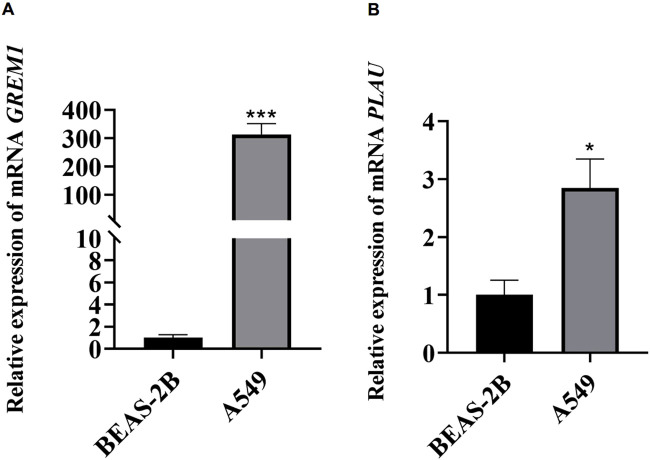
Relative mRNA expression levels of GREM1 **(A)** and PLAU genes **(B)** in BEAS-2B cell line (left, black) and A549 (right, grey). Data were means ± SEM. Experiments were repeated three times.

The RT-qPCR results showed the mRNA expression levels of the two genes (GREM1 and PLAU) involved in our IRGI risk model in LUAD cells and normal lung epithelial cells. Specifically, compared with BEAS-2B cells, the mRNA expressions of GREM1 and PLAU in A549 cells were significantly upregulated, consistent with our database analysis findings (Figure 8).

## 4 Discussion

In this study, we utilized comprehensive RNA-seq datasets and multiple bioinformatic methods to accurately predict the prognosis and immunotherapy response of LUAD patients. Our findings underscore the critical role of IRGs in survival prognosis and immunotherapy response. Based on IRGs, LUAD patients were stratified into two distinct subtypes: IRG-high and IRG-low patterns. Patients with the IRG-high pattern exhibited favorable survival outcomes. We further applied ESTIMATE and TIMER algorithms to assess immune infiltration, and demonstrated that the IRG-high patient subgroup exhibited a higher level of immune cell infiltration, including B cells, neutrophils, CD4^+^ T cells, and CD8^+^ T cells. Hence, the IRG-based subtyping could serve as a potential biomarker for prognostic risk stratification, especially through the construction of an interpretable machine learning model with robustly improved predictive performance. The IRGI risk score, which incorporates PLAU and GREM1, offers a novel approach to predicting outcomes and informing the identification of LUAD patients who are more likely to benefit from ICIs. Our results align with the growing body of literature that supports the integration of immunogenomic profiling in personalized immunotherapy for LUAD (Zhang J. et al., 2020; Skoulidis and Heymach, 2019; Zhang C. et al., 2020; Zuo et al., 2020).

To evaluate the potential immune evasion capacity and predict patient responses to immunotherapy, we utilized the TIDE analysis and revealed that patients with IRG-high pattern had significantly lower TIDE scores compared to those with IRG-low, indicating that the IRG-high patient subgroup could benefit more from ICIs. The integration of IRGs into current therapeutic strategies for LUAD patients could potentially enhance treatment efficacy (Jiang et al., 2020; Song et al., 2020). By identifying patients with different IRG patterns, clinicians could tailor immunotherapy to those most likely to respond, thereby personalizing treatment approaches. Our findings also suggest the integration of IRG expression analysis with XGBoost machine learning algorithm could notably improve the effectiveness of risk assessment and treatment decision-making. To our knowledge, this is the first research leveraging the SHAP method to optimize and interpret the bioinformatic analysis results on IRGs. Future studies should further explore the synergistic effects and clinical utility of integrating IRGs with explainable artificial intelligence techniques to refine patient management strategies (Novakovsky et al., 2023).

In addition, we constructed the IRGI risk score signature based on two pivotal genes associated with immune activation: PLAU and GREM1. PLAU is a serine protease that promotes tumor cell migration and invasion by converting plasminogen to plasmin, which facilitates extracellular matrix degradation (Li et al., 2013; Maynard et al., 2020). Its high expression is often associated with increased tumor aggressiveness and poor prognosis in cancers such as colon cancer and NSCLC (Li et al., 2013; Maynard et al., 2020). Meanwhile, PLAU may suppress anti-tumor immunity by altering the TME and facilitating immune evasion. GREM1 acts as an antagonist of the bone morphogenetic protein (BMP) signaling pathway, primarily by inhibiting BMP2 and BMP4 (Fregni et al., 2018; Liu et al., 2021; Verheyden and Sun, 2008). Aberrant expression of GREM1 could promote tumor angiogenesis, extracellular matrix remodeling, and suppression of BMP-mediated anti-tumor signaling, thereby accelerating tumor cell proliferation and migration. In our study, the functional validation of PLAU and GREM1 provided new avenues for therapeutic intervention, particularly considering the dynamic nature of TME and how it evolves in response to ICIs (Cai et al., 2020). Recent studies have highlighted the pivotal roles of PLAU and GREM1 in modulating tumor treatment responses. Under conventional therapeutic regimens, elevated PLAU expression promotes platinum-based chemotherapy resistance by enhancing DNA repair mechanisms and facilitating epithelial-mesenchymal transition (EMT) (Cui et al., 2024; Zheng et al., 2024). Furthermore, PLAU-mediated extracellular matrix (ECM) remodeling fosters a hypoxic TME, shielding cancer cells from radiation-induced apoptosis and thereby contributing to radiotherapy resistance (Bigos et al., 2024; Minchenko et al., 2014). GREM1 drives chemoresistance by antagonizing bone morphogenetic protein (BMP) signaling, thereby sustaining cancer stemness and suppressing apoptotic pathways (Yu et al., 2024; Hiroki et al., 2020). Notably, in EGFR-mutant LUAD, GREM1 overexpression perpetuates PI3K/AKT/mTOR pathway activation, sustaining tumor cell survival and proliferation, which consequently attenuates the efficacy of EGFR-TKIs such as osimertinib (Sin-Aye et al., 2020; Andreas et al., 2022). These findings posit GREM1 as a promising predictive biomarker for targeted therapy resistance. The immunomodulatory functions of these molecules hold significant implications for immunotherapy. High PLAU levels correlate with an immunosuppressive TME characterized by increased M2-like tumor-associated macrophages (TAMs) and reduced CD8^+^ T-cell infiltration—features consistently associated with poor responses to ICIs (Fan et al., 2021; Zhou et al., 2021). Similarly, GREM1 orchestrates myeloid cell polarization, promoting recruitment of M2 macrophages and regulatory T cells (Tregs), while stimulating the release of immunosuppressive cytokines (e.g., L-10, TGF-β) and impairing DC function. These mechanisms collectively diminish the therapeutic efficacy of PD-1 inhibitors (Liberty et al., 2023; Shipeng et al., 2024; Aji et al., 2025).The integration of scRNA-seq data, as demonstrated in this study, offers a promising approach to dissect the heterogeneity of LUAD and enhance our understanding of IRGs in tumor progression and response to therapy.

Our study has several limitations. The retrospective nature of and the reliance on public datasets may introduce biases that could affect the generalizability of the results, and thus further validation in larger, prospective cohorts is warranted. Meanwhile, the complexity of the immune response and the functional implications of the identified IRGs should be considered in future research. Despite these limitations, this study shed light on the development of immunogenomic-based prognostication and personalized treatment strategies for LUAD patients.

## 5 Conclusion

This study has significant clinical implications. By integrating scRNA-seq with bulk RNA-seq data to construct IRG-based model, which could serve as a reliable and independent biomarker for predicting patient outcomes and immune cell infiltration levels. We further deployed an interpretable XGBoost machine learning algorithm to robustly improve the predictive performance, allowing for more personalized risk stratification and clinical decision-making. Additionally, we identify two novel key molecules significantly associated with LUAD patient prognosis and therapeutic response to immunotherapy. IRG-based signature enhances our understanding of the heterogeneity and complexity of immune cell infiltration and function in the TME, providing valuable insights into individualized immunotherapy strategies.

## Data Availability

The original contributions presented in the study are included in the article/Supplementary Material, further inquiries can be directed to the corresponding author.

## Supplementary material

SUPPLEMENTARY FIGURE S1 Gene ontology (GO) and Kyoto Encyclopedia of Genes and Genomes (KEGG) analyses of PLAU and GREM1.

SUPPLEMENTARY FIGURE S2 The correlation between single nucleotide variants (SNVs) and copy number variations (CNVs) and the targeted PLAU and GREM1 gene expression.

SUPPLEMENTARY FIGURE S3 Transcription factors common to both PLAU and GREM1.

SUPPLEMENTARY FIGURE S4 Prediction of chemotherapeutic drugs in high-risk and low-risk groups.

SUPPLEMENTARY TABLE S1 A total of 1,181 genes significantly associated with tumor biology.

SUPPLEMENTARY TABLE S2 Twenty clusters annotated by marker genes of eight cell types.

SUPPLEMENTARY TABLE S3 PLAU and GREM1 primer sequences.
