# Supplementary figures and images for: Interpretable artificial intelligence based on immunoregulation-related genes predicts prognosis and immunotherapy response in lung adenocarcinoma
Source: Front Bioinform. 2025 Sep 19;5:1613761. doi: 10.3389/fbinf.2025.1613761 (PMC12491262; doi:10.3389/fbinf.2025.1613761)

Supplementary Figures:

Figure S1

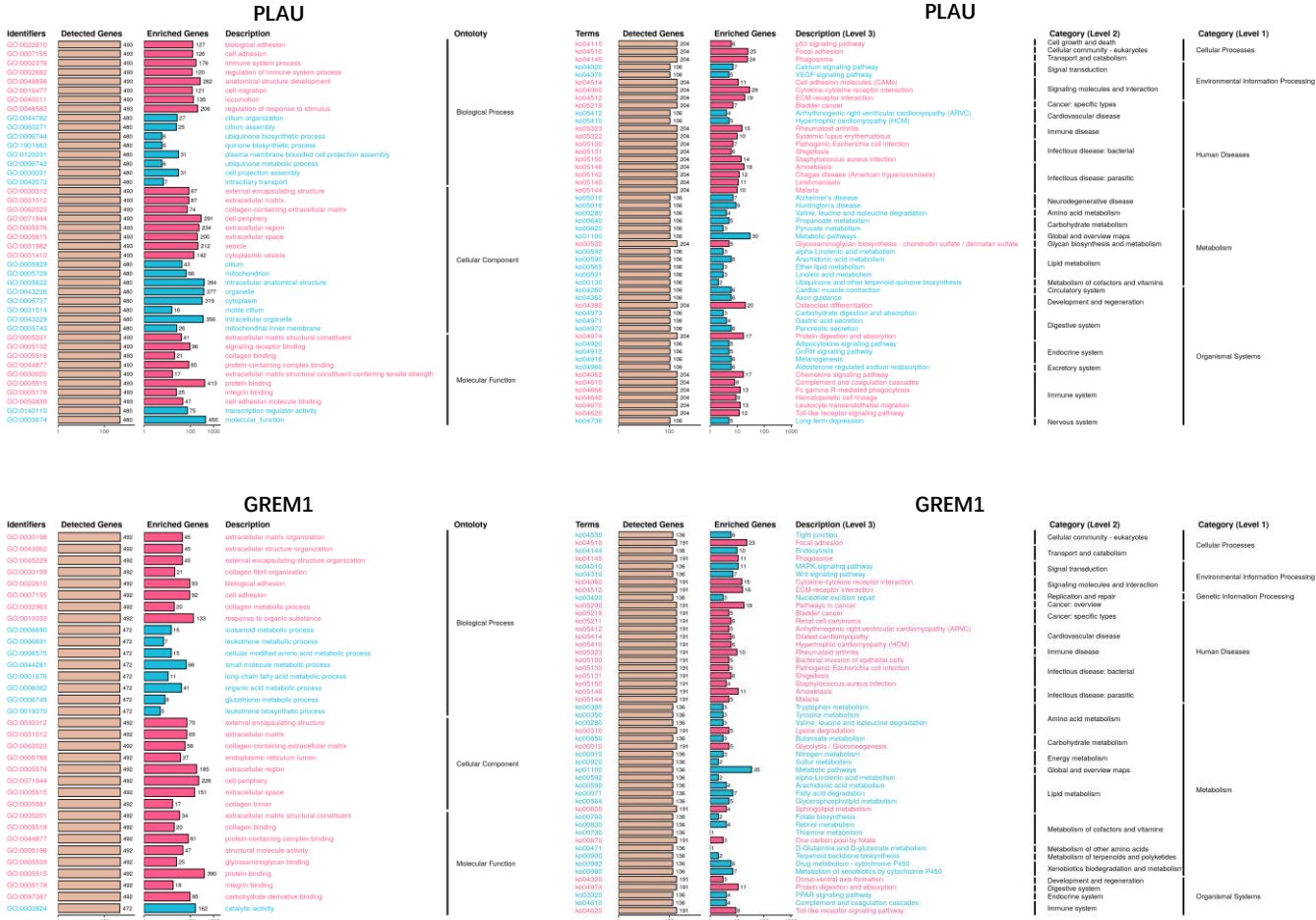

Figure S2

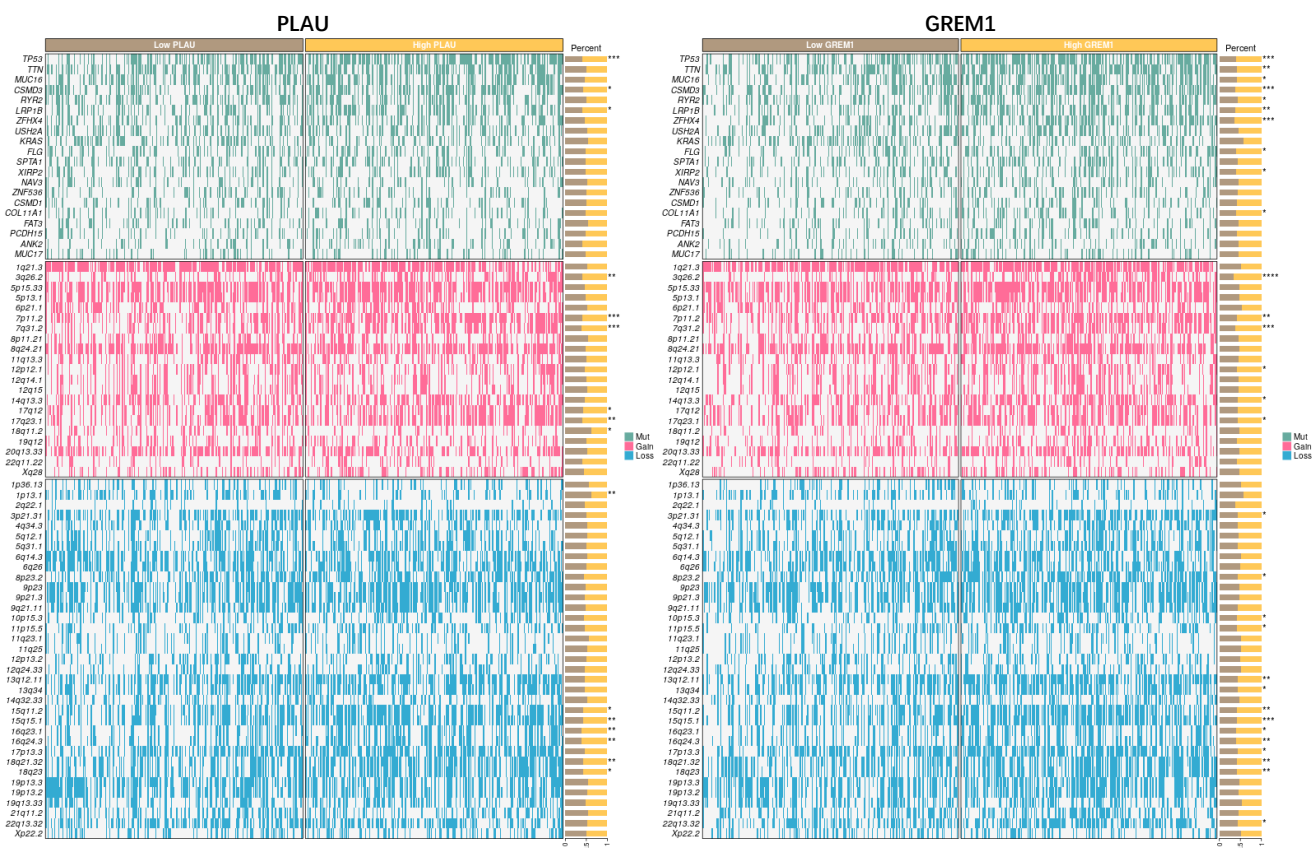

Figure S3

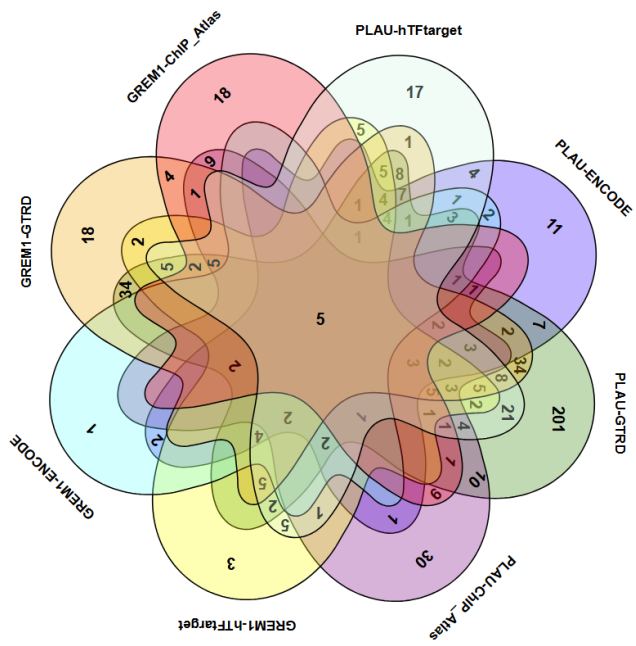

**Figure S4**

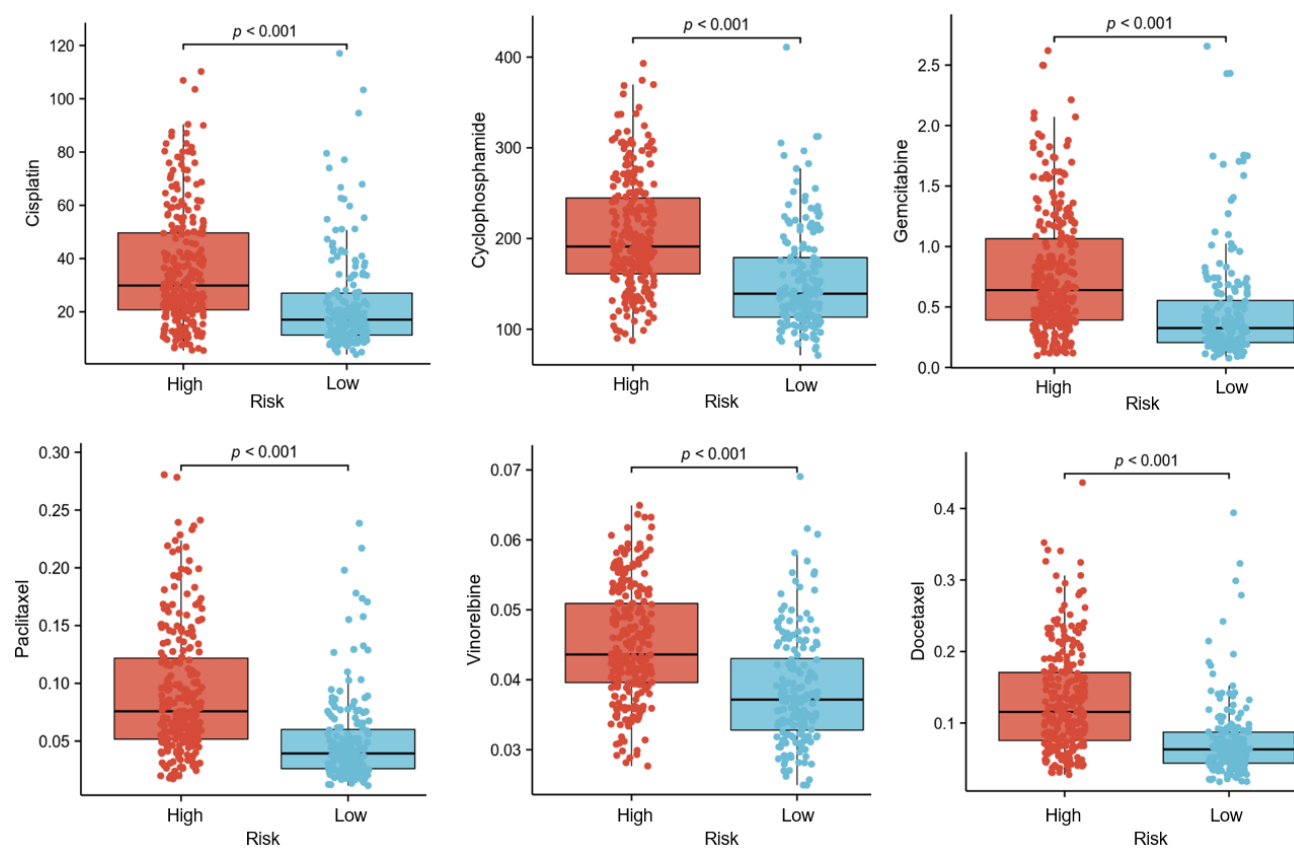

Supplement: Supplementary file 2 [file DataSheet1.pdf]
